# Long-term prognosis of preserved hearing function after surgery in patients with cerebellopontine angle tumors other than vestibular schwannoma

**DOI:** 10.1007/s00701-025-06507-6

**Published:** 2025-07-14

**Authors:** Norio Ichimasu, Michihiro Kohno, Nobuyuki Nakajima, Kyosuke Matsunaga, Ken Matsushima, Kiyoaki Tsukahara

**Affiliations:** 1 Department of Neurosurgery, Hachioji Medical Center, Tokyo Medical University, Hachioji, Tokyo Japan; 2 Department of Neurosurgery, Tokyo Medical University, 6-7-1, Nishi-Shinjuku, Shinjuku-Ku, Tokyo 160-0023 Japan; 3 Department of Otorhinolaryngology, Head and Neck Surgery, Tokyo Medical University, Shinjuku, Tokyo Japan

**Keywords:** CP angle, Hearing prognosis, Postoperative hearing function, Meningioma, Jugular foramen, Transpetrosal approach

## Abstract

**Background:**

In our previous study on vestibular schwannomas (VSs), we found that preserved useful hearing function in patients immediately after surgery gradually deteriorated in long-term period, and was lost in 13% of patients 5 years after surgery. In this retrospective study, we aimed to investigate the long-term hearing prognosis of patients with cerebellopontine angle (CPA) tumors other than VSs, and clarify whether the phenomenon of hearing deterioration after surgery occurs only in patients with VSs.

**Method:**

Patient backgrounds and otologic data were investigated in 70 patients (meningioma: 37; trigeminal schwannoma: 9; facial nerve schwannoma: 4; jugular foramen schwannoma: 9; and epidermoid cysts: 11) with preserved useful hearing function after surgery (American Academy of Otolaryngology-Head and Neck Surgery classification Class A or B).

**Results:**

Of the 70 patients (average age: 44 years; tumor diameter: 35 mm; resection rate: 96%; follow-up period: 62 months; 61 patients in Class A and 9 patients in Class B immediately after surgery), 60 patients (86%) had unchanged, 5 patients (7%) had improved, and 5 patients (7%) had worsened hearing class during the follow-up period. Only 1 patient (facial nerve schwannoma) experienced a change from Class B to C, and was out of useful-hearing range at the final follow-up (the useful hearing preservation rate: 99%). Distortion product otoacoustic emissions (DPOAEs) reflecting inner ear function were detected in 61 patients (87%) at the final follow-up, and only 4 patients (6%) demonstrated a worsening of DPOAEs during the postoperative follow-up period.

**Conclusions:**

Useful hearing function after surgery was preserved at a high rate during a long-term postoperative period in patients with typical CPA tumors other than VSs. Compared with patients with VSs, in whom hearing loss owing to inner ear dysfunction is not rare in the long-term after surgery, patients with CPA tumors demonstrated a apparently lower incidence of inner ear dysfunction and a more favorable long-term hearing prognosis. In patients with CPA tumors, not only hearing improvement by the surgery but also its long-term preservation can be expected. In this study, we confirmed that the phenomenon of postoperative hearing deterioration occurs only in patients with VSs among all CPA tumors.

## Introduction

In recent decades, the preservation of hearing function has become an important issue in the surgical treatment of all cerebellopontine angle (CPA) tumors. We previously reported that in patients with VS [6], which is the most common type of CPA tumor, hearing function that was initially preserved after surgery deteriorated over a long-term period, although there was no tumor recurrence [2–4, 6, 11–15, 18, 19, 21, 22]. In our previous retrospective study, we found that some patients with VS whose useful hearing function was preserved after surgery experienced gradual worsening of their useful hearing function from approximately 3 years after surgery. The preservation rates of their useful hearing decreased to 94%, 75%, and 65% of that immediately after surgery at 5, 8, and 10 years after surgery, respectively. Furthermore, we previously investigated the hearing function of 378 patients with CPA/intratemporal tumors other than vestibular schwannomas (VSs) who underwent surgical removal, and found that an improvement in hearing function could be expected after surgery even when the patient preoperatively had severe hearing loss [7]. In this our previous study, preoperative and postoperative hearing function was evaluated. Regarding the hearing function of patients with CPA tumors other than VS, there have only been sporadic reports limited to short-term changes, such as just before and after surgery [5, 7–9, 16, 17, 20, 23]. However, to the best of our knowledge, no reports are available on long-term postoperative outcomes for these tumors thus far. This retrospective study investigated the long-term results of hearing function in patients with CPA tumors other than VS using the auditory tests with distortion product otoacoustic emissions (DPOAEs), pure tone average (PTA) and speech discrimination score (SDS), before surgery, immediately after surgery, and at final follow-up point. Moreover, in this study we aimed to clarify whether the phenomenon of postoperative hearing deterioration is specific to patients with VSs.

## Materials and methods

### Patient selection

Of the 924 patients with CPA tumors who underwent a tumor resection during the target period from April 2013 to January 2020, we excluded patients with VS, reoperation, and neurofibromatosis type 2 (NF2), recurrence, and patients where the cochlear nerve and tumor were clearly unrelated. This study included 70 patients where useful hearing function (American Academy of Otolaryngology-Head and Neck Surgery [AAO-HNS] classification Class A or B) was preserved immediately after surgery, follow-up period is at least one year, all auditory tests (PTA, SDA, and DPOAEs) were available (37 patients of CPA meningioma [52.9%], nine patients of trigeminal schwannoma [12.9%], four patients of facial nerve schwannoma [5.7%], nine patients of jugular foramen schwannoma [12.9%], and 11 patients of epidermoid cysts [15.7%]).

### Images and auditory tests

Contrast-enhanced MRI was performed on all patients before and immediately after surgery, and at the final follow-up point. The maximum diameter of the tumor was measured in preoperative contrast-enhanced MRI axial images, and tumor extension into the internal auditory canal (IAC) was evaluated in three grades (N: none; L: less than half; M: more than half). Tumor resection rates were determined on the basis of intraoperative findings and postoperative contrast-enhanced MRI within one week after surgery. Most patients with facial nerve schwannoma undergo surgery to decompress the tumor and to preserve the facial nerve function.

Preoperative auditory tests included PTA, SDS, DPOAE, auditory brainstem responses (ABR), self-administered audiometry, and orientation testing within three months preoperatively. Postoperatively, PTA, SDS, and DPOAE were performed within three months after surgery and at the final follow-up. The measuring instruments used for PTA and SDS were AA-75 or AA-78 (RION Co., Ltd., Japan), and for DPOAE, were ILO292-USB (manufactured by RION) and ILO software, respectively.

### Surgical indications and surgical approaches

In our principle, if the patient presents with a large tumor and strong brainstem compression, surgery is indicated even if the patient is asymptomatic. Even though the tumor size is moderate or small, if it shows neurological symptoms such as facial sensory disturbance, trigeminal neuralgia, facial nerve palsy, or facial spasms due to tumor compression, or if it shows a rapid growth tendency, surgery was indicated with the consent of the patient and patient’s family after checking the risks for general anesthesia. For elderly patients, either a conservative strategy or radiotherapy was prioritized unless the tumor was large.

The surgical approach was selected based on various factors such as tumor localization, its positional relationship with the cranial nerves (CNs) 7 th and 8 th, and the purpose of the surgery. Although the retrosigmoid lateral suboccipital approach (LSO) was the first choice, in the following cases, skull-base approaches, such as the anterior transpetrosal approach (ATP), middle fossa approach (MF), retrolabyrinthine approach (RL), transmastoid approach, combined transpetrosal approach (CTP), and transjugular approach (TJ) were applied: (1) cases in which CN 7 th and 8 th were on the dorsal side of the tumor, (2) cases in which the lesion was located within both the middle and posterior cranial fossa, (3) cases in which the surgical purpose was only to decompress the facial nerve for facial nerve schwannoma, and (4) cases of jugular foramen schwannoma.

In patients with tumor extension into the IAC, our policy was to perform surgery in which the intracanalicular portion of the tumor was exposed with a high-speed diamond drill, and then resected as much as possible, to improve hearing function and prevent recurrence. And the decompression surgery of the facial nerve was performed for FNS, as facial nerve function is the highest priority for most patients who had slight or moderate facial nerve palsy.

### Surgical procedures and management

Each patient was set in an appropriate position depending on their approach, and anesthesia was maintained during the surgery using total intravenous anesthesia except for the anesthesia induction. Depending on the lesion, motor evoked potentials (MEP), sensory evoked potentials (SEP), cochlear nerve action potentials (CNAP), auditory brainstem responses (ABR), elctromyography (EMG) for trigeminal nerve, facial nerve, and eye movement were monitored [1]. For cases in which the skull base approach was applied, lumbar drainage was placed after the induction of general anesthesia to control intracranial pressure and to avoid the risk of postoperative cerebrospinal fluid leakage. In addition, to avoid neural damage, prednisolone was administered intravenously at 30–60 mg during the surgery and orally at a dose of 1–2 mg/kg/day per body weight after the surgery. Finally, the dosage was gradually tapered off until the end of the administration.

### Data analysis

Based on the results of PTA and SDS, hearing functions were evaluated with the American Academy of Otolaryngology-Head and Neck Surgery classification (AAO-HNS classification) (Class A: PTA ≤ 30 dB and SDS ≥ 70%; Class B: 30 dB < PTA ≤ 50 dB and SDS ≥ 50%; Class C: PTA > 50 dB and SDS ≥ 50%; Class D: any PTA level and SDS < 50%). Useful hearing function was defined as Class A or B. The preoperative, postoperative and follow-up hearing classes were investigated. The postoperative hearing class and the follow-up hearing class were compared to determine whether the hearing class was maintained, improved, or worsened during the postoperative follow-up period. Between two groups, hearing class maintained or improved group and hearing class worsened group (Table 2), statistic data analyses were performed using SPSS software (version 28.0; SPSS Inc., Chicago, IL, USA). Categorical and continuous variables were analyzed using Fisher's exact test and Mann–Whitney U test, respectively. A two-sided P-value of less than 0.05 was considered to indicate a statistically significant difference between two groups.

The rusults of DPOAE which reflects the inner ear function were additionally investigated immediately after surgery and at follow-up and ranked as detected, partially detected, or not detected, and a decrease of 1 rank or more was defined as the worsening of DPOAE. For patients in which DPOAE was not detected at follow-up, it was identified since which point DPOAE had been undetectable: before surgery, immediately after surgery, or at follow-up. For patients with worsened hearing class, the type of hearing impairment that occurred during the period of postoperative follow up. was determined by the otolaryngologists based on the results of the auditory tests showing the hearing loss.

## Results

The patient backgrounds of all 70 patients were as follows: mean age 44.3 years old (20–73 years old), mean tumor diameter 34.9 mm (14–72 mm), mean resection rate 96.1% (50–100%), mean follow-up period 61.5 months (22–111 months). Tumor extension into the IAC was observed in 24 patients (34.3%) (L: 19 patients; M: 5 patients), and the surgical approaches were LSO in 33 patients (47.1%), ATP in 18 patients (25.7%), MF in 2 patients (2.9%), RL in 2 patients (2.9%), CTP in 12 patients (17.1%), and TJ in 3 patients (4.3%) (Table 1).

**Table 1:** Patient characteristics and demographics

			All tumors	MG	TS	FNS	JFS	EPD
Number of patients			70	37 (52.9%)	9 (12.9%)	4 (5.7%)	9 (12.9%)	11 (15.7%)
Age (years), mean (range)			44.3	48.7	39.6	43.5	44.0	34.0
			(20–73)	(27–73)	(24–52)	(36–50)	(34–60)	(20–47)
Tumor size (mm), mean (range)			34.9	33.5	37.4	35.0	33.1	38.7
			(14–72)	(17–61)	(30–52)	(18–50)	(14–72)	(25–47)
Resection rate (%), mean (range)			96.1	96.4	98.8	74.3	98.3	99.1
			(50–100)	(75–100)	(97–100)	(50–97)	(95–100)	(98–100)
Follow-up duration (months), mean (range)			61.5	64.5	58.9	50.5	67.1	52.7
			(22–111)	(22–111)	(36–100)	(23–84)	(24–108)	(25–89)
Tumor extension into the IAC								
None			46 (65.7%)	18 (48.7%)	9 (100%)	3 (75.0%)	9 (100%)	7 (63.6%)
Less than half			19 (27.1%)	14 (37.8%)	0	1 (25.0%)	0	4 (36.4%)
More than half			5 (7.1%)	5 (13.5%)	0	0	0	0
Surgical approach								
LSO			33 (47.1%)	24 (64.9%)	0	0	6 (66.7%)	3 (27.3%)
Skull base approach			37 (52.9%)	13 (35.1%)	9 (100%)	4 (100%)	3 (33.3%)	8 (72.7%)
ATP			18 (25.7%)	8 (21.6%)	7 (77.8%)	0	0	3 (27.3%)
MF			2 (2.9%)	0	1 (11.1%)	1 (25.0%)	0	0
RL			2 (2.9%)	0	0	2 (50.0%)	0	0
CTP			12 (17.1%)	5 (13.5%)	1 (11.1%)	1 (25.0%)	0	5 (45.5%)
TJ			3 (4.3%)	0	0		3 (33.3%)	0

ATP: anterior transpetrosal approach; CTP: combined transpetrosal approach; EPD: epidermoid cyst; FNS: facial nerve schwannoma; IAC: internal auditory canal; JFS: jugular foramen schwannoma; LSO: lateral suboccipital approach; MF: middle fossa approach; MG: meningioma; TJ: transjugular approach; TS: trigeminal schwannoma; RL: retrolabyrinthine approach

### Background in each tumor (Table 1)

There were 37 patients with CPA meningioma, including five cases of petroclival meningioma. The mean age was 48.7 years old (27–73 years old), the mean tumor diameter was 33.5 mm (17–61 mm), the mean tumor resection rate was 96.4% (75%–100%), mean follow-up period 64.5 months (22–111 months), and tumor extension into the IAC occurred in 19 patients (51.4%) (L: 14; M: 5). The surgical approaches were LSO in 24 patients (64.9%), ATP in 8 patients (21.6%), and CTP in 5 patients (13.5%).

There were nine patients with trigeminal schwannoma, the mean age was 39.6 years old (24–52 years old), the mean tumor diameter was 37.4 mm (30–52 mm), the mean tumor resection rate was 98.8% (97%–100%), and mean follow-up period 58.9 months (36–100 months). None showed tumor extension into the IAC. The all surgeries were performed with skull base approaches (ATP: 7 patients [77.8%]; MF: I patient [11.1%]; CTP: I patient [11.1%].

There were four patients with facial nerve schwannoma, the mean age was 43.5 years old (36–50 years old), the mean tumor diameter was 35.0 mm (18–50 mm), the mean tumor resection rate was 74.3% (50%–97%), and mean follow-up period 50.5 months (23–84 months). One patient showed tumor extension with less than half of the IAC. The all surgeries were performed with skull base approaches (MF: I case [25.0%]; RL: 2 cases [40.0%]; CTP: I case [25.0%]).

There were nine patients with jugular foramen schwannoma, the mean age was 44.0 years old (34–60 years old), the mean tumor diameter was 33.1 mm (14–72 mm), the mean tumor resection rate was 98.3% (95%–100%), and mean follow-up period 67.1 months (24–108 months). None showed tumor extension into IAC. The surgical approaches were LSO in 6 patients (66.7%) and TJ in 3 patients (33.3%).

**Fig. 1:**
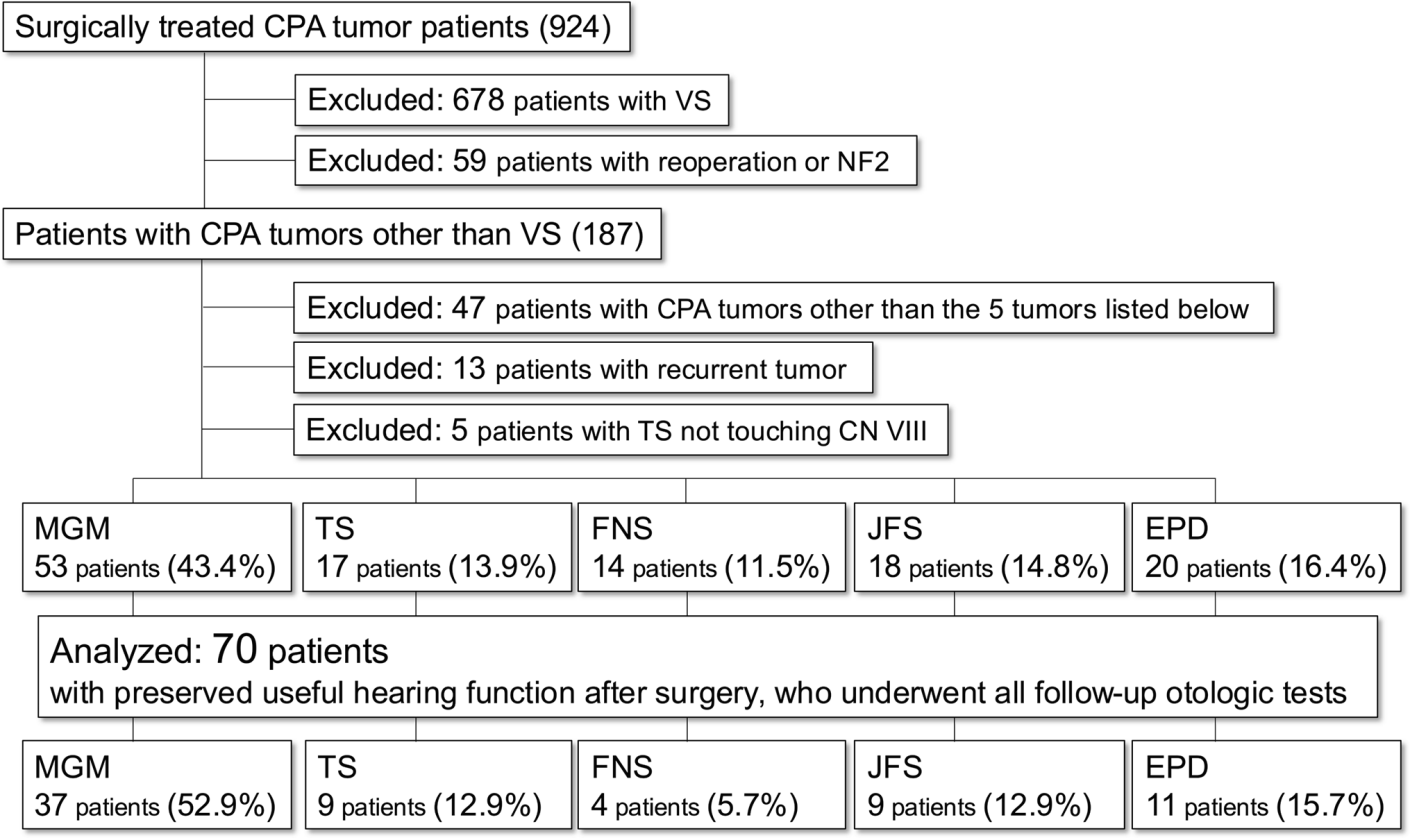
Patients selections and preoperative/postoperative hearing tests. Target patients (CPA meningioma, trigeminal schwannoma, facial nerve schwannoma, jugular foramen schwannoma, and epidermoid cyst) were extracted in the order shown in the chart. CPA: cerebellopontine angle; CN: cranial nerve; EPD: epidermoid cyst; FNS: facial nerve schwannoma; JFN: jugular foremen schwannoma; MGM: meningioma; NF2: neurofibromatosis type2; TS: trigeminal schwannoma; VS: vestibular schwannoma

There were 11 patients with epidermoid cysts, the mean age was 34.0 years old (20–47 years old), the mean tumor diameter was 38.7 mm (25–47 mm), the mean tumor resection rate was 99.1% (98%–100%), and mean follow-up period 52.7 months (25–89 months). Tumor extension into IAC was observed in four patients (36.7%) (all less than half of the IAC). The surgical approaches were LSO in 3 patients (27.3%) and skull base approaches in 8 patients (72.7%), including CTP in 5 patients (45.5%) and ATP in 3 patients (27.3%) (Fig. 1).

### Preservation of hearing classes after surgery

**Fig. 2:**
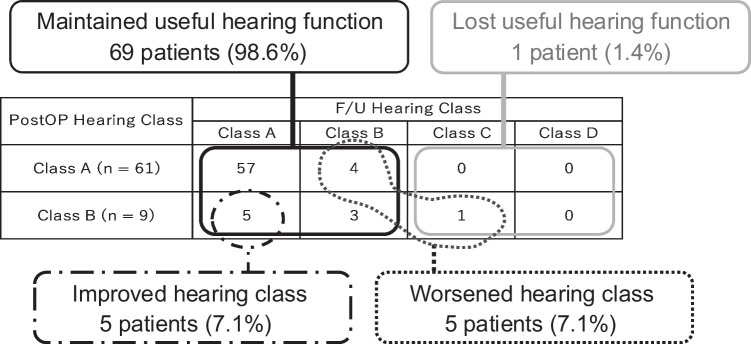
Distribution and proportion of hearing class at postoperative/final follow-up. In the target patients, 61 (87.1%) and 9 (12.9%) were in postoperative AAO-HNS classification Class A and Class B, respectively. Of the patients whose postoperative hearing class was Class A, four patients exhibited a decline to Class B at the final follow-up. Of the patients that were Class B after surgery, six patients did not exhibit any change in hearing class at the final follow-up (improved to Class A: 5 patients; worsened to Class C: I patient). AAO-HNS: American Academy of Otolaryngology-Head and Neck Surgery; F/U: follow-up; postOP: postoperative

Of the 70 target patients whose useful hearing function was preserved in the postoperative hearing test (Class A: 61 patients; Class B: 9 patients), 13 patients preoperatively had hearing loss that was out of the useful hearing range (Class C: I patient; Class D: 12 patients), however, their hearing functions were improved through surgery (CPA meningioma: 8 patients; facial nerve schwannoma: I patient; jugular foramen schwannoma: 3 patients; epidermoid cyst: I patient). During the postoperative follow up, hearing class was maintained in 60 patients (85.7%, maintained at Class A: 57 patients; Class B: 3 patients), hearing class improved (Class B to A) in five patients (7.1%), and hearing class worsened in five patients (7.1%). Of patients whose hearing class worsened, only one patient was out of the useful hearing range at the final follow-up point (Class A to B: 4 patients; Class B to C: I patient) (Fig. 2).

During the postoperative follow-up period, 65 patients were in the hearing class non-worsened group (maintained or improved group) (35 patients with meningioma, 8 patients with trigeminal schwannoma, 2 patients with facial nerve schwannoma, 9 patients with jugular foramen schwannoma, and 11 patients with epidermoid cyst). The worsening of DPOAE during the period of postoperative follow up showed a significant difference between hearing class non-worsened and worsened groups (*p* = 0.038) (Table 2). DPOAE was undetected in six patients (9.2%) at final follow-up point (undetected since the preoperative point in 3 patients). Of the five patients in which hearing class worsened during the period of postoperative follow up (2 patients with meningioma, I patient with trigeminal schwannoma, and 2 patients with facial nerve schwannoma), DPOAE was undetected at the follow-up point in three patients (60.0%) (undetected since the preoperative point in 2 patients) (Tables 2 and 3). In a survey of each patient with deteriorated hearing class, the types of hearing loss that occurred during the period of postoperative follow up were inner ear hearing loss in two patients (40.0%) and conductive hearing loss in three patients (60.0%) (Table 3). In five patients in which hearing class improved during the period of postoperative follow up (to Class A at follow-up from Class B immediately after surgery) (CPA meningioma: 4 patients, epidermoid cyst: I patient), DPOAE at follow-up was detected in all patients.

**Table 2:** Comparison of characteristics between patients with and without worsened hearing class in the follow-up auditory tests

		Hearing class		
		Maintained or improved	Worsened	*p* value
Number of patients		65 (92.9%)	5 (7.1%)	
Tumor type	MG	35 (53.8%)	2 (40.0%)	0.440
	TS	8 (12.3%)	1 (20.0%)	
	FNS	2 (3.1%)	2 (40.0%)	
	JFS	9 (13.8%)	0	
	EPD	11 (16.9%)	0	
Age (years), mean (range)		44.5 (20–73)	41.8 (36–46)	0.564
Tumor size (mm), mean (range)		35.2 (14–72)	30.4 (21–37)	0.349
Resection rate, mean (range)		96.5 (50%–100%)	90.6 (65%–100%)	0.626
IAC extension	None	41 (63.1%)	5 (100%)	0.101
	Less than half	19 (29.2%)	0	
	More than half	5 (7.7%)	0	
Worsening of DPOAE during the period of postoperative follow up		3 (4.6%)	2 (40.0%)	**0.038**
Skull base approach		34 (52.3%)	3 (60.0%)	0.555

DPOAE: distortion product otoacoustic emissions; EPD: epidermoid cyst; FNS: facial nerve schwannoma; IAC: internal auditory canal; JFS: jugular foramen schwannoma; MG: meningioma; TS: trigeminal schwannoma

**Table 3:** Characteristics of patients who experienced hearing class deterioration after surgery

Patients who maintained useful hearing function but experienced hearing class deterioration (AAO-HNS Class A to Class B)																	
Tumor type		Age	Sex	Affected side	Tumor size	Resection rate	IAC occupation	Surgical approach	Follow-up duration	AAO-HNS class			DPOAE			Change of DPOAE	Additional HL after surgery
										PreOP	PostOP	Follow-up	PreOP	PostOP	Follow-up		
Pt. 1	MG	46 years	F	Right	28 mm	100%	None	LSO	77 months	Class A	Class A	Class B	Detected	Detected	Partially detected	Worsened	Inner ear HL
Pt. 2	MG	43 years	F	Right	21 mm	100%	None	LSO	74 months	Class A	Class A	Class B	Partially detected	Partially detected	Not detected	Worsened	Inner ear HL
Pt. 3	TS	42 years	M	Left	31 mm	98%	None	ATP	36 months	Class A	Class A	Class B	Detected	Detected	Detected	Unchanged	Conductive HL
Pt. 4	FNS	36 years	F	Right	35 mm	65%	None	RL	61 months	Class A	Class A	Class B	Not detected	Not detected	Not detected	Unchanged	Conductive HL
Patient who lost useful hearing function (AAO-HNS Class C or D)																	
Pt. 5	FNS	42 yo	F	Left	35 mm	90%	None	CTP	23 months	Class C	Class B	Class C	Not detected	Not detected	Not detected	Unchanged	Conductive HL

AAO-HNS: American Academy of Otolaryngology-Head and Neck Surgery; ATP: anterior transpetrosal approach; CTP: combined transpetrosal approach; DPOAE: distortion product otoacoustic emissions; F: Female; FS: facial nerve schwannoma; HL: hearing loss; IAC: internal auditory canal; LSO: lateral suboccipital approach; M: Male; MF: middle fossa approach; MG: meningioma; postOP: postoperative; preOP: preoperative; Pt.: patient; RL: retrolabyrinthine approach; TS: trigeminal nerve schwannoma

The auditory tests at follow-up point showed that one patient (1.4%) with facial nerve schwannoma was out of the useful hearing range (Class C [PTA: 61.3 dB; SDS: 88%]). In this patient, tumor extension into IAC was not observed and the surgical approach was CTP. The result of DPOAE was undetected since the preoperative point and conductive hearing loss occurred during the follow-up period, resulting in a downgrade from Class B immediately after surgery to Class C at follow-up point (Table 3, Fig. 2).

### Analysis of DPOAEs

**Fig. 3:**
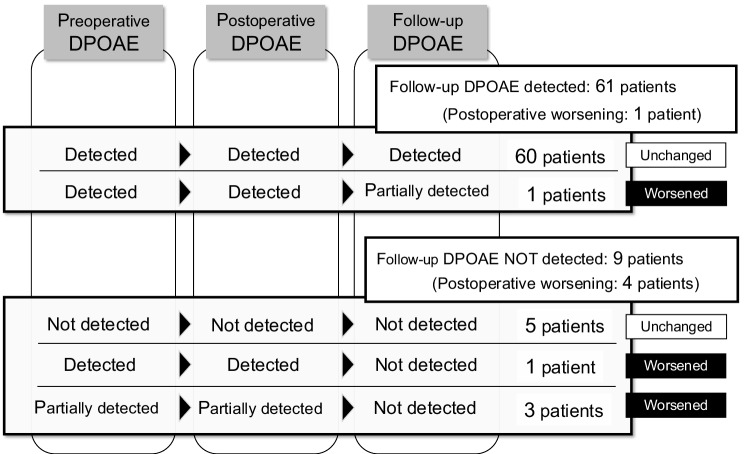
Detections of DPOAEs at three point (preoperative, postoperative, and follow-up point) and changes of DPOAEs during postoperative follow-up period. At the final follow-up point, DPOAEs were detected in 61 patients (87.1%) and undetected in 9 patients (12.9%). Of nine patients where DPOAEs were undetected, DPOAEs in five patients were undetected since preoperative point, and DPOAEs in four patients were detected immediately after surgery, but the detections either worsened or disappeared during the follow-up period. DPOAEs: distortion product otoacoustic emissions

DPOAEs were measured three times in all cases: preoperatively, postoperatively, and at final follow-up point. Comparing preoperative and postoperative DPOAEs, there were no patients where the findings changed or worsened. Preoperative DPOAEs were detected in 65 cases (92.9%) and partially detected in three of these patients (4.6%). Although the exactly same findings of DPOAE were maintained immediately after surgery, DPOAEs were undetectable in all these three patients at follow-up (Fig. 3). Follow-up DPOAEs were undetectable in nine patients (12.9%), of which five patients were undetectable since preoperative point and four patients became undetectable during the period of postoperative follow up (Fig. 3). In the hearing class non-worsened group (maintained or improved group), the findings of DPOAE worsened during the period of postoperative follow up and became undetectable in three patients (4.6%). Conversely, in the hearing class worsened group, DPOAE worsened in two patients (40.0%) (Table 2).

### Association between patients’ hearing prognosis and IAC extension of tumors

Statistical analyses showed that there was no difference in the IAC extension of tumors between the hearing class non-worsened group and the worsened group (*p* = 0.101) (Table 2). A detailed analysis of the association between DPOAEs and the IAC extension of tumors demonstrated that there was no IAC extension in 4 of the 5 patients in whom DPOAEs were not detected before surgery, and only 1 patient had a tumor occupying more than half of the IAC (*p* = 0.323). No significant association was observed between the IAC extension of tumors and the worsening of DPOAE during the postoperative follow-up period (*p* = 0.370).

### Types of additional hearing loss in patients with hearing class worsened during the period of postoperative follow up

In four patients (5.7%), the hearing class worsened within the useful hearing range (Class A to B), although useful hearing was maintained. The types of additional hearing loss for them were inner ear disfunction in two patients (Pt. 1 and 2) and conductive hearing loss in two patients (Pt. 3 and 4). For both patients with conductive hearing loss, the skull base approaches were used. As mentioned above, one patient (1.4%) was out of the useful hearing range due to conductive hearing loss occurring during the period of postoperative follow up (Pt. 5). (Table 3).

## Discussion

In vestibular schwannoma as the most common CPA tumor, there have been reports that preserved hearing function after surgery deteriorates over a long period [2–4, 11–15, 18, 19, 21, 22] and we previouslly reported that 56% of patients experienced a decline of hearing class and 13% of patients lost their useful hearing function [6] in the average follow-up period of 5.3 years. Meanwhile, favorable auditory outcomes have been reported in surgical treatments for CPA tumors other than VSs [5, 7–9, 16, 17, 20, 23]. However, the observations in those reports were short-term and limited. This retrospective study was significant because the long-term auditory function was examined in patients with these tumors with an average follow-up period of 5.2 years; 7% of patients experienced a decline of hearing class, and only 1% exhibited a loss of useful hearing function. In this study, we reported that a long-term hearing prognosis with CPA/intratemporal tumors other than VSs is quite contrastive to that with VSs. To understand the reason for this, differences between VS and other CPA tumors were discussed using the results of auditory tests effectively.

Comparing this study with our previous report on VSs [6], we found that inner ear function is an essential factor determining postoperative hearing prognosis in VSs and other CPA tumors. An inner ear function was evaluated with DPOAE, which measures the reaction of outer hair cells in the inner ear. Referring to our previous report on VSs with an average follow-up period of 5.3 years, 52.0% (27/52 patients) of patients whose useful hearing function was preserved exhibited undetectable DPOAEs at final follow-up point, and a decline in hearing class due to inner ear dysfunction was observed in 56% of the patients [6]. Meanwhile, this study on CPA tumors other than VSs showed that DPOAEs postoperatively worsened in 6.2% (4/65 patients) of patients, and the rate of hearing class worsening was 7%. These numbers indicate evidently lower incidence than in patients with VS. Moreover, the worsening of DPOAE during the period of postoperative follow up showed a significant deference with hearing class worsening (*p* = 0.038), though the number is not large enough for statistic analysis. The consideration of each patient also showed that inner ear dysfunction added after surgery was one of the reasons of postoperative hearing loss (Table 3). The CPA tumors other than VSs differ from VSs in that they are less likely to have lesions within the IAC and tend to exhibit pure retrocochlear hearing loss [7]. Long-term hearing loss after surgery for VSs is possibly related to the manipulation of lesions in the IAC, as these tumors almost always have lesions in the IAC^23^. VSs are likely to cause postoperative inner ear dysfunction. However, other types of CPA tumors, which rarely affect into the IAC, should theoretically be less likely to cause inner ear dysfunction. This hypothesis is the most understandable and reasonable to explain the difference in hearing prognosis between patients with VS and those with other types of CPA tumors, although a significant difference was not observed between IAC extension by the tumor and preservation of hearing class (*p* = 0.101). In this study, we also could not show a significant correlation between tumor extension into the IAC and DPOAEs, and none of the patients had lesions extending into the IAC in the patient group with worsened hearing class. Therefore, the mechanism by the postoperative inner ear dysfunction in two of the patients is unclear.

Consideration of each patient in this study revealed that conductive hearing loss were another reason for postoperative hearing loss (Table 3). A decline in hearing class due to the postoperative additional conductive hearing loss was observed in three patients (Pt. 3–5 in Table 3). In all these patients, the skull base approaches were used, so a scar formation around the tympanic cavity could occur and cause conductive impairment. Two of the three patients with conductive hearing loss were cases of facial nerve schwannoma. Our previous reports showed that facial nerve schwannomas are less likely to improve hearing function after surgery and tend to worsen hearing function. However, since conductive hearing loss is common in patients with facial nerve schwannoma, severe hearing loss is rare [7]. Long-term postoperative observation for patients with preserved useful hearing after surgery revealed that only 7% of patients had postoperative hearing loss, and only one patient lost the useful hearing function worsening from Class B to Class C due to conductive impairment, and none of the patients exhibited a decline to Class D.

To summarize our researches [6, 7, 10], improving hearing function with surgery for VSs is rare and challenging [10], and even if useful hearing function is preserved postoperatively, it is not guranteed that hearing class and useful hearing are maintained in the long term [6]. Contrastingly, in other types of CPA tumors, hearing function is more likely to be improved with surgery [7], and this study confirmed that hearing function more likely to be maintained over a long period. Therefore, for these tumors, maximum effort must be made to preserve hearing function through various measures, such as approach selection, neuro-monitoring settings, and gentle manipulation around the cochlear nerve.

### Limitations

The limitations of this study are that it is a retrospective study, and the number of patients is small for some of the statistical analyses. Although each CPA tumor type has distinct characteristics, a variety of CPA tumors were included in this study, owing to their rarity and to enable a comparison with VSs.

## Conclusion

This study confirmed that hearing function is maintained over a long period in typical CPA tumors other than VS. Hearing improvement can also be expected after surgery for these tumors. We confirmed that the phenomenon of postoperative hearing deterioration occurs only in patients with VSs among all CPA tumors.

## Data Availability

No datasets were generated or analysed during the current study.

## Abbreviations

AAO-HNS: American Academy of Otolaryngology-Head and Neck Surgery
ABR: Auditory brainstem responses
ATP: Anterior transpetrosal approach
CN: Cranial neve
CPA: Cerebellopontine angle
CTP: Combined transpetrosal approach
DPOAEs: Distortion product otoacoustic emissions
EPD: Epidermoid cyst
FNS: Facial nerve schwannoma
HL: Hearing loss
IAC: Internal auditory canal
JFS: Jugular foramen schwannoma
L: Less than half
LSO: Lateral suboccipital approach
M: More than half
MF: Middle fossa approach
MG: Meningioma
NF2: Neurofibromatosis type2
postOP: Postoperative
preOP: Preoperative
Pt.: Patient
PTA: Pure tone average
RL: Retrolabyrinthine approach
SDS: Speech discrimination score
TJ: Transjugular approach
TS: Trigeminal schwannoma
VS: Vestibular schwannoma
